# Comparative Genome-Centric Analysis of Freshwater and Marine ANAMMOX Cultures Suggests Functional Redundancy in Nitrogen Removal Processes

**DOI:** 10.3389/fmicb.2020.01637

**Published:** 2020-07-07

**Authors:** Muhammad Ali, Dario Rangel Shaw, Mads Albertsen, Pascal E. Saikaly

**Affiliations:** ^1^Water Desalination and Reuse Center (WDRC), Biological and Environmental Science & Engineering (BESE), King Abdullah University of Science and Technology (KAUST), Thuwal, Saudi Arabia; ^2^Center for Microbial Communities, Aalborg University, Aalborg, Denmark

**Keywords:** biological nitrogen removal, anammox, *Candidatus* Scalindua, saline wastewaters, genome-resolve, nanopore sequencing, *Candidatus* Brocadia

## Abstract

There is a lack of understanding of the interaction between anammox bacteria and the flanking microbial communities in both freshwater (non-saline) and marine (saline) ecosystems. Here, we present a comparative genome-based exploration of two different anammox bioreactors, through the analysis of 23 metagenome-assembled genomes (MAGs), 12 from freshwater anammox reactor (FWR), and 11 from marine anammox reactor (MWR). To understand the contribution of individual members to community functions, we applied the index of replication (iRep) to determine bacteria that are actively replicating. Using genomic content and iRep information, we provided a potential ecological role for the dominant members of the community based on the reactor operating conditions. In the non-saline system, anammox (*Candidatus* Brocadia sinica) and auxotrophic neighboring bacteria belonging to the phyla *Ignavibacteriae* and *Chloroflexi* might interact to reduce nitrate to nitrite for direct use by anammox bacteria. Whereas, in the saline reactor, anammox bacterium (*Ca*. Scalindua erythraensis) and flanking community belonging to phyla *Planctomycetes* (different than anammox bacteria)—which persistently growing in the system—may catabolize detritus and extracellular material and recycle nitrate to nitrite for direct use by anammox bacteria. Despite different microbial communities, there was functional redundancy in both ecosystems. These results signify the potential application of marine anammox bacteria for treating saline N-rich wastewaters.

## Introduction

Microbial communities are an abundant natural resource that represents functional biological entities with diverse metabolic capacities. Various strategies have been used to optimize the management of microbial resources to tailor the needs of specific applications (Rittmann et al., 2006). Biological wastewater treatment systems are the most common large-scale examples of these strategies where microbes remove organic and inorganic pollutants, including ammonium (${\text{NH}}_{4}^{+}$), from wastewaters. Anaerobic ammonium-oxidizing (anammox) bacteria capable of oxidizing ammonium to dinitrogen (N_2_) gas using nitrite (${\text{NO}}_{2}^{-}$) as a terminal electron acceptor are considered as one of the most energy-efficient biological nitrogen removal technologies for the treatment of ${\text{NH}}_{4}^{+}$-rich wastewater streams (Ali and Okabe, 2015).

Besides, anammox process is a critical part of the global nitrogen cycle and is detected in nearly all anoxic ecosystems containing fixed nitrogen (Humbert et al., 2010). Anammox bacteria are responsible for up to 50% of the biogeochemical N_2_ production in different ecosystems including marine environments (Dalsgaard et al., 2005, 2013; Xi et al., 2016), deep-sea hydrothermal vents (Byrne et al., 2009), marine seagrass (Garcias-Bonet et al., 2018), groundwater (Moore et al., 2011), paddy soils (Zhu et al., 2011), lakes (Schubert et al., 2006), and estuaries (Trimmer et al., 2003). More than 30 anammox species, divided among five candidate genera within the Planctomycetes phylum have been detected in non-saline engineered and natural ecosystems, and marine (saline) environments (Ali and Okabe, 2015). The anammox bacteria belonging to genera *Kuenenia, Brocadia, Anammoxoglobus*, and *Jettenia* predominantly found in freshwater ecosystems and the bacteria belonging to genus *Scalindua* are primarily found in marine ecosystems (Sonthiphand et al., 2014). Distinct partitioning of anammox bacterial communities among natural and engineered ecosystems have been observed, and it has been postulated that the global distribution pattern of anammox bacteria is governed primarily by salinity (Sonthiphand et al., 2014), their kinetic characteristics (Dale et al., 2009; Sonthiphand et al., 2014), and better aggregation ability (Ali et al., 2018).

Metagenomic analysis has been used to understand distant partitioning of anammox bacterial communities using draft genome encoded genetic content of the species. Previous metagenomic sequencing efforts have yielded draft near-complete genome assemblies of eight different anammox species (Gori et al., 2011; Speth et al., 2012, 2017; van de Vossenberg et al., 2013; Oshiki et al., 2015; Ali et al., 2016a, 2019; Park et al., 2017), and one complete genome assembly for *Ca*. Kuenenia stuttgartiensis (Frank et al., 2018). Some comparative metagenomics studies have been performed on separately enriched anammox communities (Speth et al., 2012), different microbial aggregates sizes (Guo et al., 2016), and lab-scale reactor configurations (Bhattacharjee et al., 2017) under non-saline conditions. Similarly, metagenomics studies have also been performed for saline environments in natural marine ecosystems (van de Vossenberg et al., 2013; Speth et al., 2017). However, these aforementioned studies did not adopt a genome-centric metagenomics approach but instead relied on a global analysis of genes and pathways. Genome-centric metagenomics approach enables us to infer actual contribution of certain genome(s) to a specific metabolic pathway.

There is no pure culture available for any anammox species. Previous studies indicated that uncultured members of the phyla *Chlorobi, Chloroflexi*, and *Ignavibacteriae* are omnipresent in freshwater anammox bioreactors (Ali et al., 2016b; Bagchi et al., 2016). Consequently, it is always questionable to what extent there is syntrophy between anammox bacteria and neighboring microbial communities in engineered and natural ecosystems (Lawson et al., 2017). A genome-centric metagenome approach was employed to gain a comprehensive insight into the function of the entire microbial community in various non-saline anammox bioreactors (Speth et al., 2016; Lawson et al., 2017). Typically, anammox bioreactors contain mixed microbial communities. These studies enhanced our understanding on metabolite exchange reactions pertaining to nitrogen cycle (nitrate recycling) and interaction of anammox bacteria with autotrophic nitrifying bacteria (Speth et al., 2016) and heterotrophic bacteria (Lawson et al., 2017) in non-saline anammox-based bioreactors. Marine anammox bacteria could be a promising alternative for the treatment of ${\text{NH}}_{4}^{+}$-rich saline industrial and/or municipal wastewater (Ali et al., 2020). Previously, we reported the enrichment of anammox culture in a membrane bioreactor (MBR) capable of treating to treat ${\text{NH}}_{4}^{+}$-rich wastewater under moderate salinity and in the presence of organic carbon (Ali et al., 2020). However, little is known about the microbial community and the syntrophic cooperation between the microbial community of saline anammox-based bioreactors.

We, therefore, hypothesized that even though marine and freshwater environments harbor totally different communities, we expect to find functional redundancy in the nitrogen removal process in saline and non-saline anammox reactors. Functional redundancy enables functional resistance of an ecosystem to environmental perturbations due to the presence of multiple species that can perform the same metabolic function (e.g., phosphorous removal, ammonium oxidation, nitrite oxidation, etc.) such that the loss of one species due to perturbation will be substituted by another species in the community (Saikaly and Oerther, 2011; Louca et al., 2018). In this study, we mainly focused on functional redundancy in terms of nitrogen removal processes of phylogenetically distant anammox bacteria in saline and non-saline ecosystems. To test this hypothesis, we performed a genome-centric metagenome analysis of two physiologically distant (i.e., freshwater and marine) anammox-based bioreactors. Four months' time-series short-read sequencing data of both cultures were *de novo* assembled, and used for differential coverage binning to identify contigs belonging to the same microbial genome (Albertsen et al., 2013; Nielsen et al., 2014). It is a challenge to assemble a complete or closed genome from a metagenome sequencing data provided by 2nd generation sequencing platforms (e.g., Illumina). Biases and artifacts introduced during inherent DNA amplification steps often lead to fragmented genome coverage. Besides, the relatively short read lengths prevent the resolution of large genomic repeats, highly similar paralogs, and other structural variations by the assembler, leaving the assembly incomplete (Eid et al., 2009). Repeat elements constrain the assembly contiguity of the genomes assembled from short-read sequencing data. In contrast, 3rd generation sequencing technologies, for instance, the Oxford Nanopore DNA sequencing, do not require DNA amplification and generate long reads that facilitates the recovery of complete or near-complete genomes with one or just a few scaffolds. Particularly, nanopore sequencing technology is free of context-specific biases and removes the potential biases introduced through PCR amplification (Eid et al., 2009; Flusberg et al., 2010). Previously, Single-Molecule Real-Time (SMRT) sequencing, a 3rd generation sequencing technology, was used to sequencing the closed genome of anammox species *Ca*. Kuenenia Stuttgartiensis from a highly enriched (>95%) culture growing as suspended planktonic cells in a membrane bioreactor (Frank et al., 2018). However, the study of anammox bioreactors containing complex microbiome using long-read sequencing technology is lacking. Therefore, we applied nanopore sequencing and workflow, which incorporates nanopore long-read assembly and Illumina short-read correction, to assemble uninterrupted complete or near-complete metagenome-assembled genomes (MAGs) from complex microbiome residing in both bioreactors. Further, to understand the role of individual microbiome members, we used peak-to-noise coverage ratio method, index of replication (iRep) (Brown et al., 2016), to calculate microbial population replication rates in both bioreactors. A putative functional role was assigned to each MAG based on reactor performance and bacterial growth. The resulting population genomes were used to construct system-wide ecological models for both bioreactors.

## Materials and Methods

### Origin of Anammox Cultures

Freshwater anammox was harvested as granular biomass from a continuous up-flow column bioreactor (Tsushima et al., 2007). Marine anammox biomass was harvested as a biofilm attached to a non-woven fabric sheet, from an up-flow column bioreactor (Kindaichi et al., 2011). Both reactors were fed with synthetic wastewater as mentioned elsewhere (van de Graaf et al., 1995). An artificial sea salt SEALIFE (Marine Tech, Tokyo, Japan) was supplemented into the media to a final concentration of 30 g L^−1^ (3% salinity) in the marine anammox reactor.

### Establishment and Operation of Reactors

Two 1-liter up-flow column reactors (XK 50/60 Column, GE Healthcare, UK) were established (Figure S1). One reactor was inoculated with granular anammox biomass dominated by *Ca. Brocadia sinica* (hereafter referred as Freshwater Reactor, FWR) and another reactor was inoculated with anammox biofilm attached to a non-woven fabric sheet with culture dominated by *Ca*. Scalindua (hereafter referred to as Marine Water Reactor, MWR). Both reactors were fed with synthetic medium that contained; ${\text{NH}}_{4}^{+}$ (2.5–20) mM, ${\text{NO}}_{2}^{-}$ (2.5–24) mM, CaCl_2_ 100 mg L^−1^, MgSO_4_ 300 mg L^−1^, KH_2_PO_4_ 30 mg L^−1^, KHCO_3_ 500 mg L^−1^ and trace element solutions (van de Graaf et al., 1995). The synthetic medium was prepared with deionized water for FWR, and with fresh Red Sea water for MWR. Hydraulic retention time (HRT) was set at 0.15 (FWR) and 0.46 days (MWR). Important operating parameters for both reactors were described in Table S1. During reactor operation, concentrations of ${\text{NH}}_{4}^{+}$, ${\text{NO}}_{2}^{-}$, and ${\text{NO}}_{3}^{-}$ in the influent and effluent of the reactors were measured using a spectrophotometric method (Eaton et al., 2005) in multilabel plate readers (SpectraMax Plus 384; Molecular Devices, CA, USA).

### Library Preparation and DNA Sequencing

Biomass samples were collected from both reactors at 0 (inoculum), 2, and 4 months of operation. The DNA was extracted using the Fast DNA spin kit for soil (MP Biomedicals, Tokyo, Japan) according to the manufacturer's instructions. The DNA was quantified using Qubit (Thermo Fisher Scientific, USA), and 50 ng DNA was used to prepare Illumina Nextera libraries following the manufacturer's instructions (Illumina, USA). The DNA library was paired-end sequenced (2 × 250 bp) using shotgun sequencing on a Hiseq 2500 system (Illumina, USA).

For nanopore sequencing, one sample from each reactor was chosen. The sample chosen was 0 for FWR and 2 months for MWR because of high anammox bacteria abundance in these samples. DNA size distributions were visualized with Genomic DNA ScreenTapes on the Agilent 2200 TapeStation system (Agilent, USA). The library was prepared using the SQK-LSK109 protocol in which samples were barcoded with the EXPNBD104 kit and pooled in an approximately equimolar ratio. The library (~50 fmole) was loaded onto a primed FLO-MIN106D flow cell with 1,339 available pores and sequenced in MinKnow Release 19.06.7. Fast5 files were basecalled in Guppy v. 3.1.5 using the Flip-Flop algorithm.

### Bioinformatics Processing

The sequence reads were trimmed for Nextera adaptors using cutadapt (v. 1.10; Martin, 2011) with a minimum phred score of 20 and a minimum length of 150 bp. The trimmed reads were assembled using spades (v. 3.7.1; Bankevich et al., 2012). The reads were mapped back to the assembly using BWA (v. 0.7.15-r1142-dirty; Li and Durbin, 2010) to generate coverage files for metagenomic binning. Open reading frames (ORFs) were predicted in the assembled scaffolds using Prodigal (Hyatt et al., 2010). A set of 117 hidden Markov models (HMMs) of essential single-copy genes were searched against the ORFs using HMMER3 (http://hmmer.janelia.org/) with default settings, with the exception that option (*-cut_tc*) was used (Dupont et al., 2012). Identified proteins were taxonomically classified using BLASTP against the RefSeq (v.52) protein database with a maximum e-value cut-off of 10^−5^. MEGAN was used to extract class-level taxonomic assignments from the BLAST output (Tamura et al., 2011). All the subsequent data processing was performed using the step-by-step guide (http://madsalbertsen.github.io/mmgenome/) for differential coverage binning (Albertsen et al., 2013).

Similarly, for nanopore raw reads, basecalled data was subsequently demultiplexed, and adaptors were trimmed in Porechop v. 0.2.4. Filtlong v. 0.2.0 was used to filter the data with min_length set to 7,000 bp. Trimmed nanopore reads were assembled using Canu (v. 1.9.0; Koren et al., 2017). The raw assembly was subjected to various polishing steps. Polishing consisted of processing with minimap (v. 2.15.0; Li, 2018), followed by racon (v. 1.4.0; Vaser et al., 2017), followed by two iterations of medaka (v. 0.8.0; Oxford Nanopore Technologies, 2018) to polish contigs with the nanopore reads. Subsequently, raw Illumina reads were mapped against polished nanopore contigs, and later racon was finally used to polish contigs with the Illumina reads.

The supporting data for binning was generated according to the description in the mmgenome package (v. 2.0.14; Karst et al., 2016). Metagenome binning was carried out in R (v. 3.3.1; R Core Team, 2013) using the R-studio environment. The MAGs bins were manually refined as described in the mmgenome package, and the final MAGs were annotated using PROKKA (v. 1.12-beta; Seemann, 2014). Predicted amino acid sequences (amino acid sequences in FASTA format produced by PROKKA) were annotated by KOALA (KEGG Orthology And Links Annotation) for K number assignment of KEGG Genes (Kanehisa et al., 2016). The quality of recovered MAGs was assessed using CheckM (v. 1.0.9; Parks et al., 2015) and the MAGs were classified as high-quality, medium-quality, or low-quality based on the guidelines provided elsewhere (Bowers et al., 2017). Further, annotated genome assemblies (GFF3 format produced by PROKKA) were processed by Roary (Page et al., 2015) for comparative genome analysis. An algorithm, index of replication (iRep), was applied to determine which bacteria are actively replicating in the system (Brown et al., 2016). All the MAGs with a minimum average coverage of 10 were subjected to the measurement of replication using default parameters.

### Phylogenomics Analysis

The taxonomic affiliation of the recovered MAGs was accomplished using Anvi'o version 2.4.0 (Eren et al., 2015), based on a set of ribosomal proteins (Campbell et al., 2011). The closely related genomes of the recovered MAGs were identified using Genome Taxonomy Database (GTDB) as described elsewhere (Chaumeil et al., 2020). The similar ribosomal proteins were also identified in publicly available genomes closely related to the recovered MAGs and used to build a phylogenetic tree. Ribosomal proteins were aligned and concatenated in Anvi'o following the instructions described in the workflow for phylogenomics. Then, a neighbor-joining phylogenetic tree was constructed with concatenated ribosomal proteins in MEGA7 (Kumar et al., 2016).

## Results

### Reactor Performance

The two bioreactors showed stable performance during the study period (Figure S2). Total inorganic nitrogen removal efficiency was about ~80% after inoculation and remained consistent throughout the duration of the experiment with Nitrogen Removal Rate (NRR) of 3.0 (FWR) and 0.3 kg-N m^−3^ d^−1^ (MWR). Stoichiometric ratios of consumed ${\text{NO}}_{2}^{-}$ to consumed ${\text{NH}}_{4}^{+}$ (Δ${\text{NO}}_{2}^{-}$/Δ${\text{NH}}_{4}^{+}$) and produced ${\text{NO}}_{3}^{-}$ to consumed ${\text{NH}}_{4}^{+}$ (Δ${\text{NO}}_{3}^{-}$/Δ${\text{NH}}_{4}^{+}$) were in the range of 1.0–1.5 and 0.12–0.23, respectively. Those stoichiometric ratios are close to the theoretical ratios of anammox reaction (i.e., 1.15 for Δ${\text{NO}}_{2}^{-}$/Δ${\text{NH}}_{4}^{+}$ and 0.16 for Δ${\text{NO}}_{3}^{-}$/Δ${\text{NH}}_{4}^{+}$) (Lotti et al., 2014), indicating that anammox was mainly responsible for nitrogen removal in both reactors.

### General Genome Features

The genome DNAs were extracted from both reactors after 0, 2, and 4 months, and deeply sequenced to obtain a system-wide view of the microbial community. The combined metagenome assembly, produced from Illumina reads (all three samples), generated a total of 15,020 contigs with N50 of 19,646 bp for FWR and 19,253 contigs with N50 of 30,841 bp for MWR. The metagenome assembly produced from nanopore sequencing data, corrected with Illumina short-reads, generated a total of 1,293 contigs with N50 of 382,194 bp for FWR and 3,690 contigs with N50 of 72,430 bp for MWR. These high-quality assemblies allowed us to extract 23 MAGs of the most abundant microbes, 12 in FWR, and 11 in MWR (Table 1). These MAGs were recovered using differential coverage plots generated from separate coverage files (Figures S3, S4).

**Table 1:** Characteristics of the Metagenome-assembled genomes (MAGs) obtained in this study.

**MAG ID**	Isolation source^*^	**Technology**	**Phylum**	**MAG size (Mbp)**	**No. contigs**	**Completeness (%)**	**Contamination (%)**	**Quality**	**Accession no**.
AMX1	FWR	Nanopore + Illumina	Planctomycetes	4.06	2	96.7	1.65	High	JABFFU01
IGN2	FWR	Nanopore + Illumina	Ignavibacteriae	3.86	1	99.44	1.12	High	CP053446
IGN3	FWR	Nanopore + Illumina	Ignavibacteriae	3.74	1	94.41	0	High	CP053447
CHB4	FWR	Illumina	Chlorobi	2.72	72	88.39	6.01	Medium	JABFFV01
BCD5	FWR	Illumina	Bacteroidetes	2.53	55	82.26	0.54	Medium	JABFFW01
ATM6	FWR	Illumina	Armatimonadetes	2.73	15	92.28	0.93	High	JABFFX01
ATM7	FWR	Illumina	Armatimonadetes	2.39	55	76.39	0.93	Medium	JABFFY01
CFX8	FWR	Illumina	Chloroflexi	5.51	158	83.82	3.64	Medium	JABFFZ01
CFX9	FWR	Illumina	Chloroflexi	2.94	173	76.36	0.36	Medium	JABFGA01
CFX10	FWR	Illumina	Chloroflexi	5.55	76	98.48	0.00	High	JABFGB01
CFX11	FWR	Nanopore + Illumina	Chloroflexi	4.62	2	79.7	1.82	Medium	JABFGC01
CFX12	FWR	Nanopore + Illumina	Chloroflexi	4.28	3	81.15	0	Medium	JABFGD01
AMX13	MWR	Nanopore + Illumina	Planctomycetes	4.74	4	96.59	3.41	High	JABFGE01
CLD14	MWR	Illumina	Calditrichaeota	5.33	29	99.94	4.40	High	JABFGF01
CFX15	MWR	Illumina	Chloroflexi	5.13	44	91.82	3.64	High	JABFGG01
IGN16	MWR	Illumina	Ignavibacteriae	4.94	77	99.72	1.96	High	JABFGH01
PRO17	MWR	Illumina	Proteobacteria	3.05	20	97.54	1.28	High	JABFGI01
BCD18	MWR	Illumina	Bacteroidetes	3.27	24	100.00	0.00	High	JABFGJ01
PRO19	MWR	Nanopore + Illumina	Proteobacteria	3.49	1	97.74	3.82	High	CP053448
PRO20	MWR	Nanopore + Illumina	Proteobacteria	2.67	1	92.27	3.04	High	CP053449
PNC21	MWR	Nanopore + Illumina	Planctomycetes	4.55	2	96.59	1.14	High	JABFGK01
PNC22	MWR	Nanopore + Illumina	Planctomycetes	4.12	1	90.34	0	High	CP053450
PRO23	MWR	Nanopore + Illumina	Proteobacteria	2.91	1	70.7	2.35	Medium	CP053451

* *FWR, Freshwater Reactor; MWR, Marine Water Reactor*.

**Figure 1:**
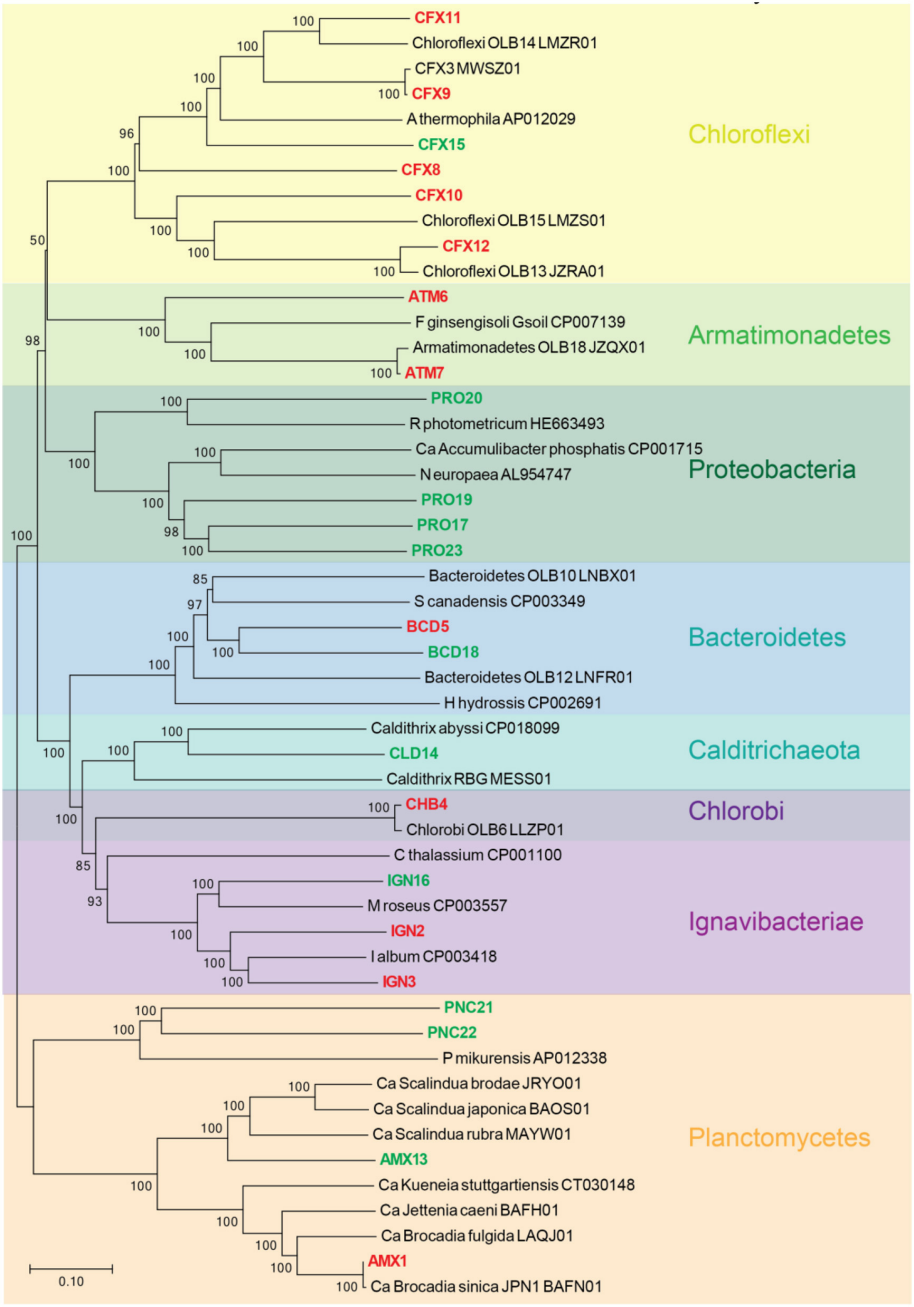
Neighbor-joining phylogenetic tree showing the evolutionary relationship between all recovered metagenome-assembled genomes (MAGs) and the closely related genomes downloaded from the NCBI genome repository. The closely related genomes were identified using Genome Taxonomy Database (GTDB) as described elsewhere (Chaumeil et al., 2020). Tree includes MAGs recovered from freshwater (red) and marine water (green) reactor. GenBank accession numbers for each genome are provided. Branch node numbers represent bootstrap support values and the bootstrap consensus inferred from 1,000 iterations.

A phylogenetic tree was constructed from the recovered population genomes and the reference genomes based on a set of concatenated ribosomal protein sequences (Figure 1). The MAGs recovered from FWR represent organisms from the phyla Armatimonadetes (2 MAGs), Bacteroidetes (1 MAGs), Chlorobi (1 MAGs), Chloroflexi (5 MAGs), Iganavibacteriae (2 MAGs), and Planctomycetes [1 MAG, identified as anammox bacterium *Ca*. Brocadia sinica, Average Nucleotide Identity (ANI) 99.99%]. The MAGs extracted from MWR belong to Bacteroidetes (1 MAG), Calditrichaeota (1 MAG), Chloroflexi (1 MAG), Iganavibacteriae (1 MAG), Proteobacteria (4 MAGs), and Planctomycetes (3 MAGs including an anammox bacterium affiliated with the genus *Ca*. Scalindua as expected from a saline ecosystem). The marine anammox MAG has 99.9% nucleotide-level genomic similarity with previously sequenced anammox bacterium (“*Ca. Scalindua* sp.,” NCBI accession number RBMW01) (Ali et al., 2019). This microbial genome (RBMW01) was assembled from short-read sequencing data, and the assembly has 121 contigs. We proposed the name of this species as “*Ca*. Scalindua erythraensis” [etymology: G. adj. *erythraensis*; from the Erythraean sea (Red Sea)]. The marine anammox bacterium was enriched in an up-flow biofilm reactor treating real Red Sea wastewater, and therefore Red Sea was used for the species name. The majority of other genomes obtained in this study also represent novel microbes with no cultured or sequenced representatives, emphasizing the lack of knowledge on the microbial community in these types of ecosystems.

### Microbial Community Abundance

**Figure 2:**
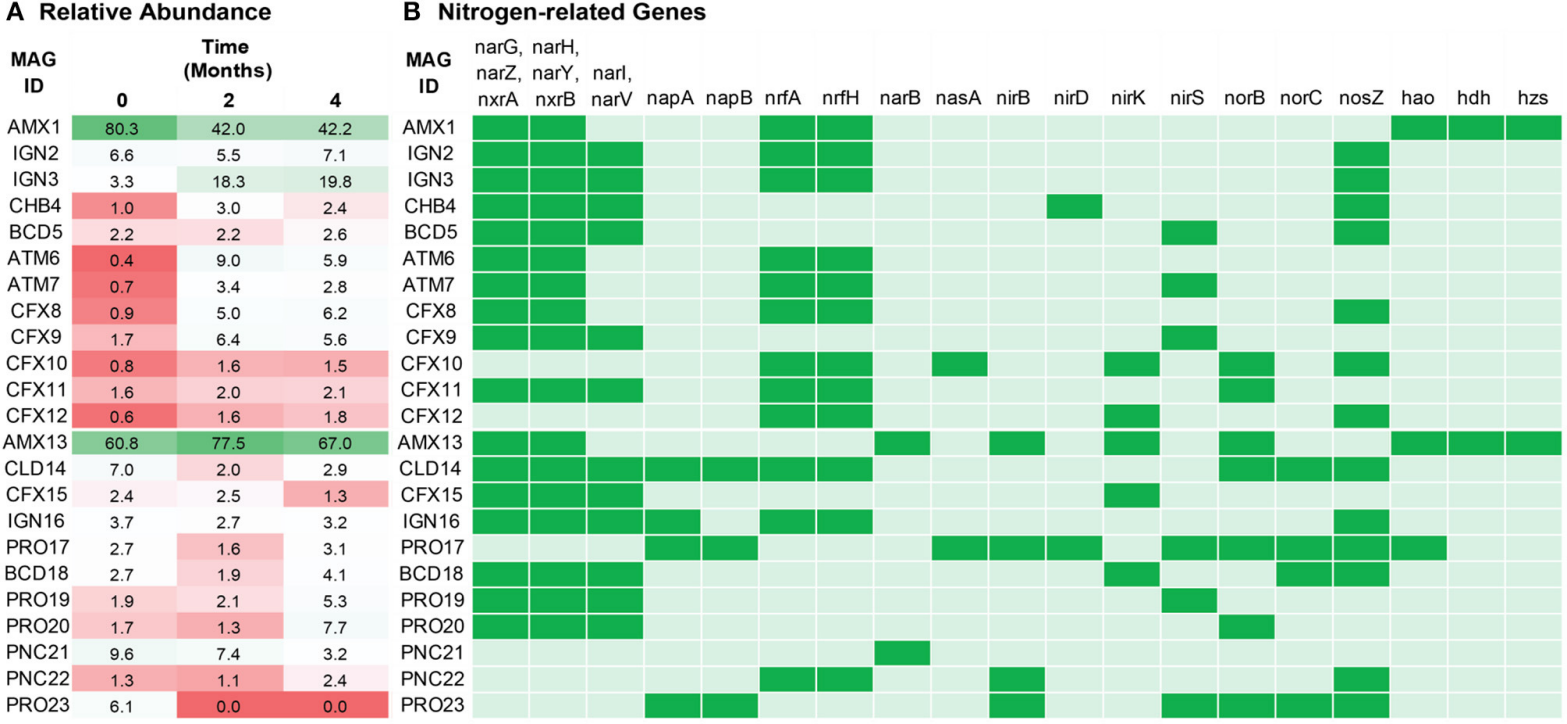
**(A)** Relative abundance of the dominant organisms in freshwater (top) and marine water (bottom) reactor. Anammox bacteria (AMX1 and AMX13) were the most dominant bacteria in both ecosystems. Relative abundance is based on reads per million values of metagenomics reads that mapped to each MAG. **(B)** The presence/absence analysis of nitrogen related marker genes from the draft genomes annotated by KOALA (KEGG Orthology And Links Annotation). Green indicates the presence and light green indicate the absence of the marker genes.

The relative abundance of extracted MAGs was estimated based on their coverage in the metagenomes. In total, the relative abundance of these recovered MAGs accounted for more than 90% of the quality-filtered sequencing reads for both reactors, which indicates that the sequencing depth provided sufficient resolution to obtain a comprehensive insight into the microbial ecology of these bioreactors. The relative read abundance of each MAG was for simplicity treated as reflective of the abundance of that organism in each reactor (Figure 2A). However, the readers are reminded that the observed relative abundances might have been affected by differences between organisms in terms of DNA extraction efficiency (Albertsen et al., 2015) and GC sequencing bias. As expected, the most abundant bacteria in both reactors belonged to anammox, having a coverage about two orders of magnitude higher as compared to the other MAGs. In FWR, *Ca*. Brocadia sinica population dropped from about 80–42% within the first 2 months. The overall population of the FWR changed drastically from 0 to 2 months of operation. For example, bacterial population belonging to IGN3, ATM6, CFX8, and CFX9 significantly increased from 3, 0, 1, and 2% to 18, 9, 5, and 6%, respectively. The dynamic change in the population structure of the FWR might be an effect of adaption to the new medium compared to the inoculum. The second most dominant organisms (IGN2 and IGN3) in FWR (3–20%) were closely related to *Ignavibacterium album*, which has previously been reported as the second most dominant phylum in previous studies conducted on non-saline anammox bioreactors (Ali et al., 2016b; Bhattacharjee et al., 2017; Lawson et al., 2017). The MAGs belonging to the phylum Armatimonadetes and Chloroflexi were the other dominant members of the bacterial community in the FWR.

In contrast, the MWR responded differently, with *Ca*. Scalindua being further enriched from 61 to 78% in the first 2 months of operation. Interestingly, the phylum Ignavibacteriae was not among the dominant member of the microbial community in MWR compared to FWR. The second most abundant MAG in the MWR (PNC21) belonged to the phylum Planctomycetes (family *Phycisphaeraceae*), which suggests that they might have an important ecological role in the reactor.

### Nitrogen Turnover in the Bioreactors

The reactors were continuously fed with inorganic synthetic wastewater (${\text{NH}}_{4}^{+}$ and ${\text{NO}}_{2}^{-}$-rich). Hence, analysis of the recovered population MAGs was focused on genes encoding key enzymes of the nitrogen cycle (Table S2): hydroxylamine oxidoreductase (*hao*), nitrate reductase (*nar* and *nap*), and nitrate oxidoreductase (*nxr*) for interconversion of nitrite and nitrate; nitrite reductase (*nirK* and *nirS*), nitric oxide reductase (*norBC* and *norZ*), and nitrous oxide reductase (*nos*) for denitrification; pentaheme nitrite reductase (*nrf*) for respiratory ammonification; and hydrazine synthase (*hzs*) and hydrazine dehydrogenase (*hdh*) for anammox metabolism. As expected, anammox bacteria encoded the key enzymes responsible for core anammox metabolism, e.g., *nxr, hzs*, and *hdh* (Figure 2B).

The central metabolic pathways of anammox organisms have previously been described (Kartal et al., 2011) through a metagenome from an enrichment culture of *Ca. Kuenenia stuttgartiensis* (Strous et al., 2006). Previously recovered genomes led to the identification of three redox reactions essential for anammox catabolism: (1) reduction of ${\text{NO}}_{2}^{-}$ to nitric oxide (NO) via nitrite reduction enzyme (NIR), (2) condensation of ${\text{NH}}_{4}^{+}$ and NO in hydrazine by HZS enzyme, and (3) oxidation of hydrazine into di-nitrogen gas mediated by HDH enzyme. The Anammox genomes recovered in the present study (AMX1 and AMX13) encoded the key enzymes responsible for the core anammox reaction. However, the nitrite reduction gene (*nirS* or *nirK*) was not detected in AMX1, indicating this bacterium might reduce ${\text{NO}}_{2}^{-}$ through some novel enzyme. The nitrite reduction gene (*nirS* or *nirK*) encoded in the flanking microbial population genomes (BCD5, 18, AMT7, CFX9, 10, 12, and 15, PRO17, 19, and 23), are possibly used for detoxification to cope with fluctuating nitrite levels in the reactors. Many extracted genomes lack the *nor* gene that could also supply NO to anammox and the flanking bacterial population. Also, from the whole flanking population, only CFX10, PRO17, BCD18, and PRO23 encoded a complete set of denitrification enzymes responsible for the conversion of nitrate (or nitrite) to dinitrogen gas. This suggests that partial denitrification and exchange of N-cycle intermediates could play an essential role in both systems. Similar incomplete denitrification pathways were also observed in genome-resolved metagenomics studies on engineered (Speth et al., 2016) and natural ecosystems (Baker et al., 2015). Together, these functions could facilitate a nitrite loop with anammox bacteria and consequently enhance overall nitrogen removal performance in the bioreactor.

Detection of nitrous oxide (N_2_O) has frequently been reported in lab-scale (Okabe et al., 2011; Ali et al., 2016b) and full-scale anammox reactors (Kampschreur et al., 2008; Castro-Barros et al., 2015; Harris et al., 2015). N_2_O accounts for about 6% of all global greenhouse gas emissions from human activities and has 300 times higher global warming potential as compared to carbon dioxide (Ciais et al., 2013). In FWR, none of the population genomes encoded NOR enzymes, except for CFX11. Nevertheless, population genomes such as AMX13, and PRO20 genomes encoded NOR enzyme without *nos*, suggesting that these microbes could mediate the release of N_2_O in MWR or could be used for detoxification of NO to N_2_O. It is worth mentioning that there are some population genomes (IGN2-3, CHB4, CFX8, CFX12, IGN16, and PNC22) containing *nos* gene which could utilize N_2_O from bulk solution and reduce it to dinitrogen gas.

**Figure 3:**
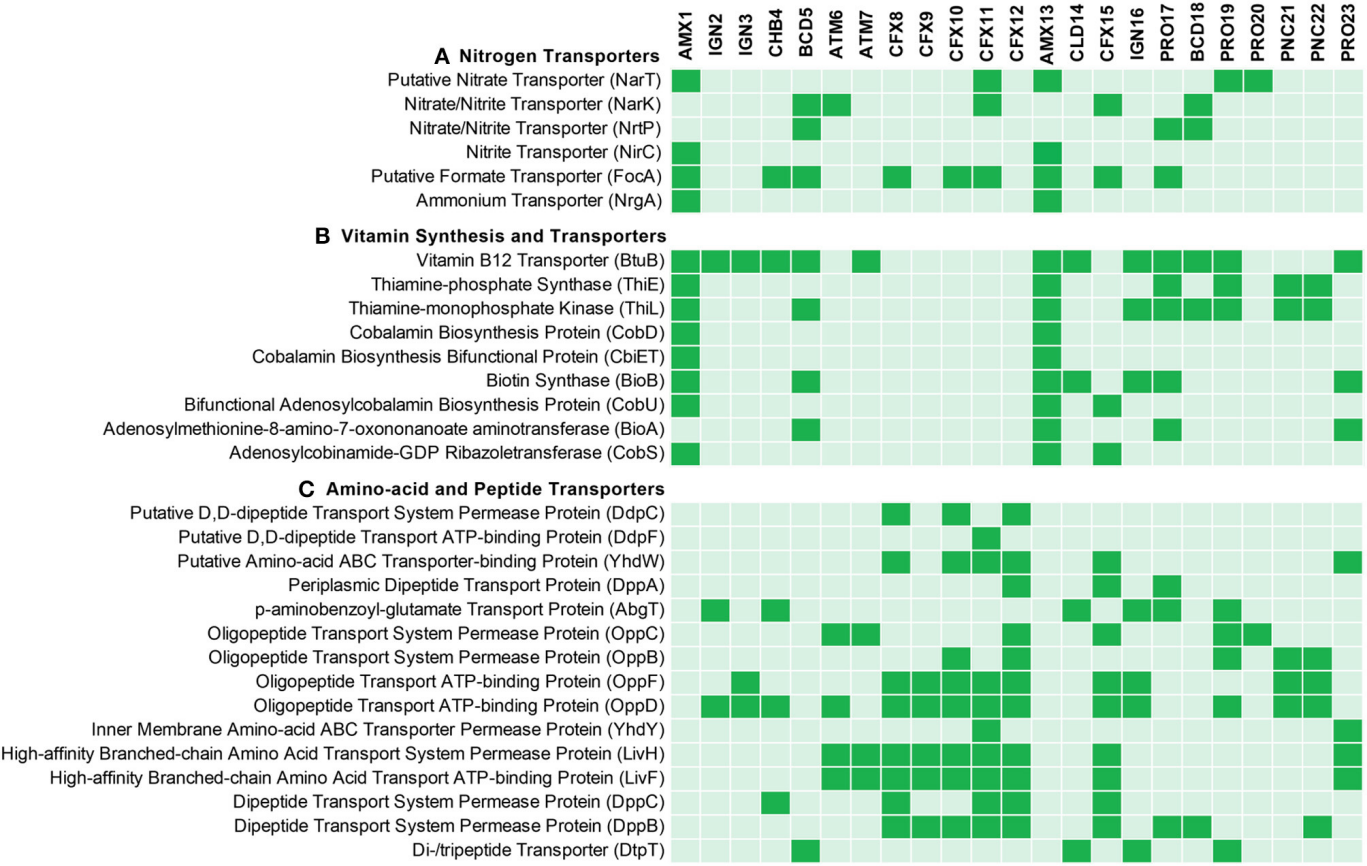
Predicted **(A)** nitrogen related transporters, **(B)** vitamin synthesis and transporters and **(C)** amino acid and peptide transporters predicted across the recovered MAGs. The genomes were annotated using PROKKA and annotated genomes were processed by Roary for comparative analysis.

Ammonium is one of the most abundant forms of nitrogen in wastewaters. Therefore, each MAG was evaluated for the genes that encode membrane proteins involved in the transport of nitrogen species to further understand the nitrogen metabolism in the members of the community (Figure 3A). Ammonium transporter genes encoding transmembrane ammonium transport proteins (NRGA) were solely present in the anammox genomes (AMX1 and AMX13), underlying the importance of anammox bacteria for removal of ammonium in anoxic environments.

In natural environments, ${\text{NO}}_{2}^{-}$ is the least abundant form of inorganic nitrogen and could be the limiting factor for anammox bacteria in biofilms. The anammox genomes contained genes (*focA/nirC*) encoding transporter proteins from the Formate/Nitrite Transporter (FNT) family. The structural analysis of the formate transporter revealed that the protein assembles into a homo-pentamer, which might act as a channel instead of active transport, and mediates high-flux transport of nitrite across the inner membrane (Wang et al., 2009). Also, ammonium and FNT transporter genes were not identified in the neighboring community members.

Nitrate is another stable form of inorganic nitrogen in natural and engineered ecosystems. Nitrate is an end-product of the anammox reaction and it is produced form the oxidation of ${\text{NO}}_{2}^{-}$ to ${\text{NO}}_{3}^{-}$. The AMX1 and AMX13 genomes contains genes encoding for nitrate transporter enzyme (NART). This enzyme is involved in the uptake and transport of nitrate into cells, with the subsequent reduction to ${\text{NO}}_{2}^{-}$ and/or ${\text{NH}}_{4}^{+}$, catalyzed by the enzymes nitrate reductase (NAR) and/or nitrite reductase (NRF), respectively (Kartal et al., 2007; Ali et al., 2015; Oshiki et al., 2016b). However, contrary to AMX1, the AMX13 genome lacked the *nrf* gene. Instead, AMX13 genome contains a gene (*nirB*) encoding for nitrite reduction, which could mediate the six-electron reduction of ${\text{NO}}_{2}^{-}$ to ${\text{NH}}_{4}^{+}$. In general, ${\text{NH}}_{4}^{+}$ is not a limiting substrate in an anoxic natural marine environment. Instead, ${\text{NO}}_{2}^{-}$ is often the limiting substrate, which could be synthesized from ${\text{NO}}_{3}^{-}$ by anammox bacteria under limiting substrate conditions. It should be noted that many neighboring community members contain genes encoding for dissimilatory nitrate reduction to ammonium (DNRA) (Figure 2B), which could also facilitate supply of ${\text{NO}}_{2}^{-}$ and/or ${\text{NH}}_{4}^{+}$ by reduction of ${\text{NO}}_{3}^{-}$. It is also pertinent to mention that anammox bacteria can reverse NXR enzymatic reaction, which then can mediate reduction of ${\text{NO}}_{3}^{-}$ back to ${\text{NO}}_{2}^{-}$ in the presence of organics (Kartal et al., 2007; Oshiki et al., 2013; Ali et al., 2020), thus supplying ${\text{NO}}_{2}^{-}$ to anammox bacteria.

### Transport of Vitamins, Amino Acids, and Peptides

Metabolite exchange of vitamins, peptides, and amino acids are known to shape microbial community assembly (Mee et al., 2014; Hubalek et al., 2017). Interestingly, anammox and most of the neighboring population genomes encoded the gene (*btuB*) for the vitamin B12 transporter (Figure 3B). However, it was observed that most of the abundant flanking population were missing the critical genes involved in thiamin (vitamin B1) and biotin (vitamin B7) biosynthesis. None of the neighboring population genomes encoded genes for the synthesis of cobalamin (vitamin B12), while B-vitamin biosynthesis genes were encoded in the anammox genomes (AMX1 and AMX13), suggesting that anammox bacteria may support B-vitamin requirements for members of the flanking community in both bioreactors. The breakdown of extracellular polymeric substances (EPS) produced by anammox and other bacteria has been suggested to be a primary source of organic carbon for heterotrophic bacterial growth in anammox bioreactors (Hou et al., 2015; Liu et al., 2016; Lawson et al., 2017). The flanking community in the reactors (mainly, belonging to phylum Ignavibacteriae, Chlorobi, and Chloroflexi) encoded proteins required for transport of peptide and amino acids (Figure 3C), which may suggest that these flanking community members have been selected to degrade peptides, amino acids and EPS produced by anammox bacteria.

### Carbon Fixation Pathways

**Figure 4:**
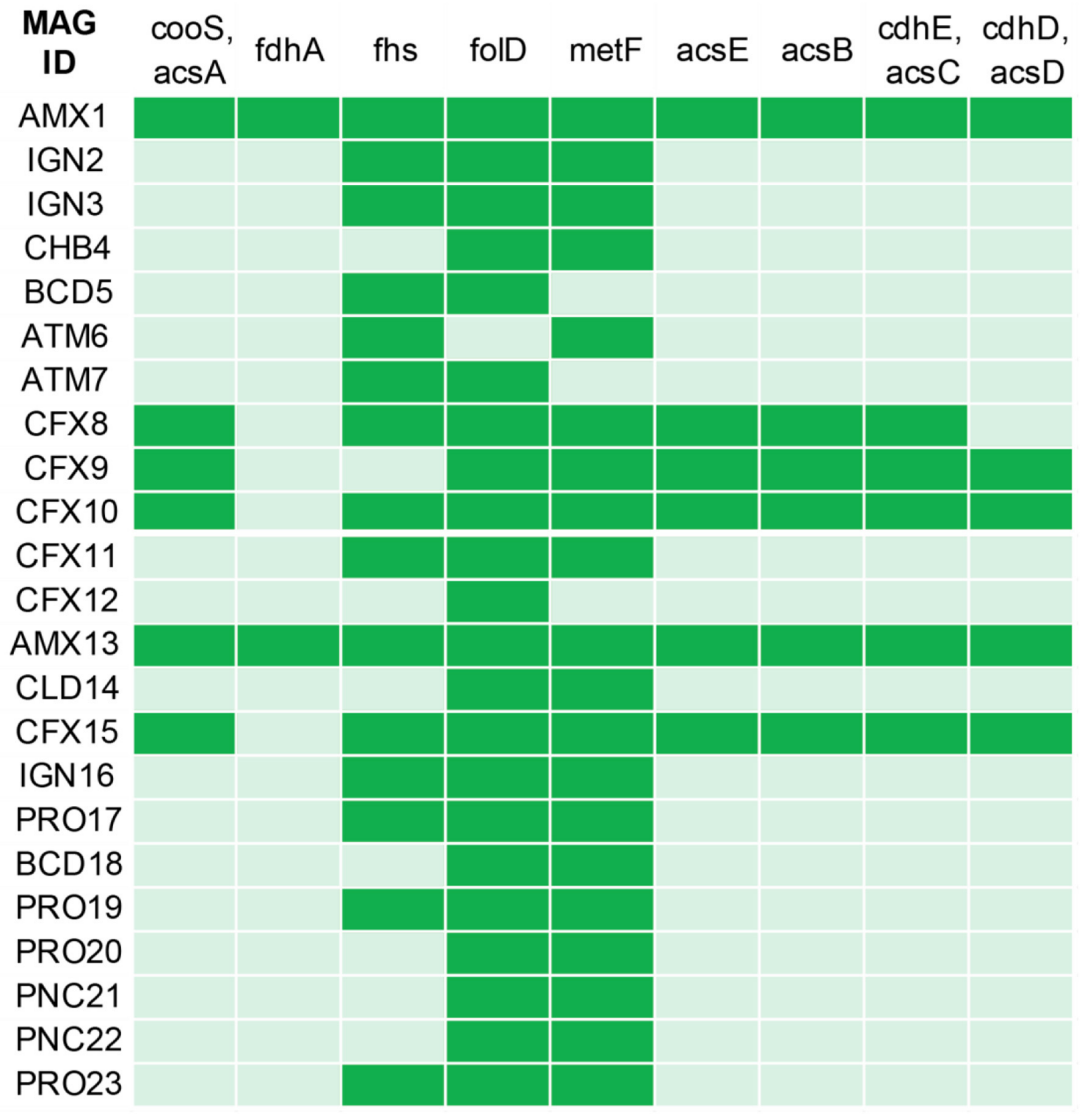
Marker genes involved in the biochemical CO_2_ fixation pathway (Wood–Ljungdahl pathway) predicted across the recovered MAGs annotated by KOALA (KEGG Orthology And Links Annotation). The genes encoding key enzymes involved in Wood–Ljungdahl pathway: anaerobic carbon-monoxide dehydrogenase catalytic subunit (cooS, acsA); formate dehydrogenase (NADP+) alpha subunit (fdhA); formate–tetrahydrofolate ligase (fhs); methylenetetrahydrofolate dehydrogenase (NADP+)/methenyltetrahydrofolate cyclohydrolase (folD); methylenetetrahydrofolate reductase (NADPH) (metF, MTHFR); 5-methyltetrahydrofolate corrinoid/iron sulfur protein methyltransferase (acsE); acetyl-CoA synthase (acsB); acetyl-CoA decarbonylase/synthase, CODH/ACS complex subunit gamma (cdhE, acsC); acetyl-CoA decarbonylase/synthase, CODH/ACS complex subunit delta (cdhD, acsD). Green indicates the presence and light green indicate the absence of the marker genes.

CO_2_ fixation is another core metabolic pathway in anammox bacteria, where CO_2_ is assimilated using the reductive acetyl-coenzyme (Acetyl-CoA) pathway (Strous et al., 2006), commonly known as Wood–Ljungdahl pathway. Specific enzymes (CO Dehydrogenase and acetyl-CoA synthase) catalyze the reaction for the conversion of CO to acetyl-CoA. CO_2_ fixation using the reductive acetyl-CoA requires reducing power, which is obtained from the ${\text{NO}}_{2}^{-}$ oxidation to ${\text{NO}}_{3}^{-}$ by the NXR enzyme (Kartal et al., 2013). Both anammox genomes (AMX1 and AMX13) contained genes encoding for CO dehydrogenase/acetyl-CoA synthase involved in the reductive acetyl-CoA pathway, suggesting that both bacteria perform CO_2_ fixation via the reductive acetyl-CoA pathway coupled with ${\text{NO}}_{2}^{-}$ oxidation (Figure 4). Interestingly, MAGs belonging to the phylum Chloroflexi (CFX8-10 and CFX15) also contained genes encoding for the key enzymes required for CO_2_ fixation via the reductive acetyl-CoA pathway that would permit a mixotrophic growth. Furthermore, the population genomes CFX8, CFX10, and CFX15 can obtain energy required for CO_2_ fixation via ${\text{NO}}_{2}^{-}$ oxidation to ${\text{NO}}_{3}^{-}$ similar to anammox bacteria. However, these MAGs also have the ability to use alternative electron donors such as H_2_ and organic carbon (derived from detritus and extracellular material).

### Measurement of Bacterial Replication Rates

**Figure 5:**
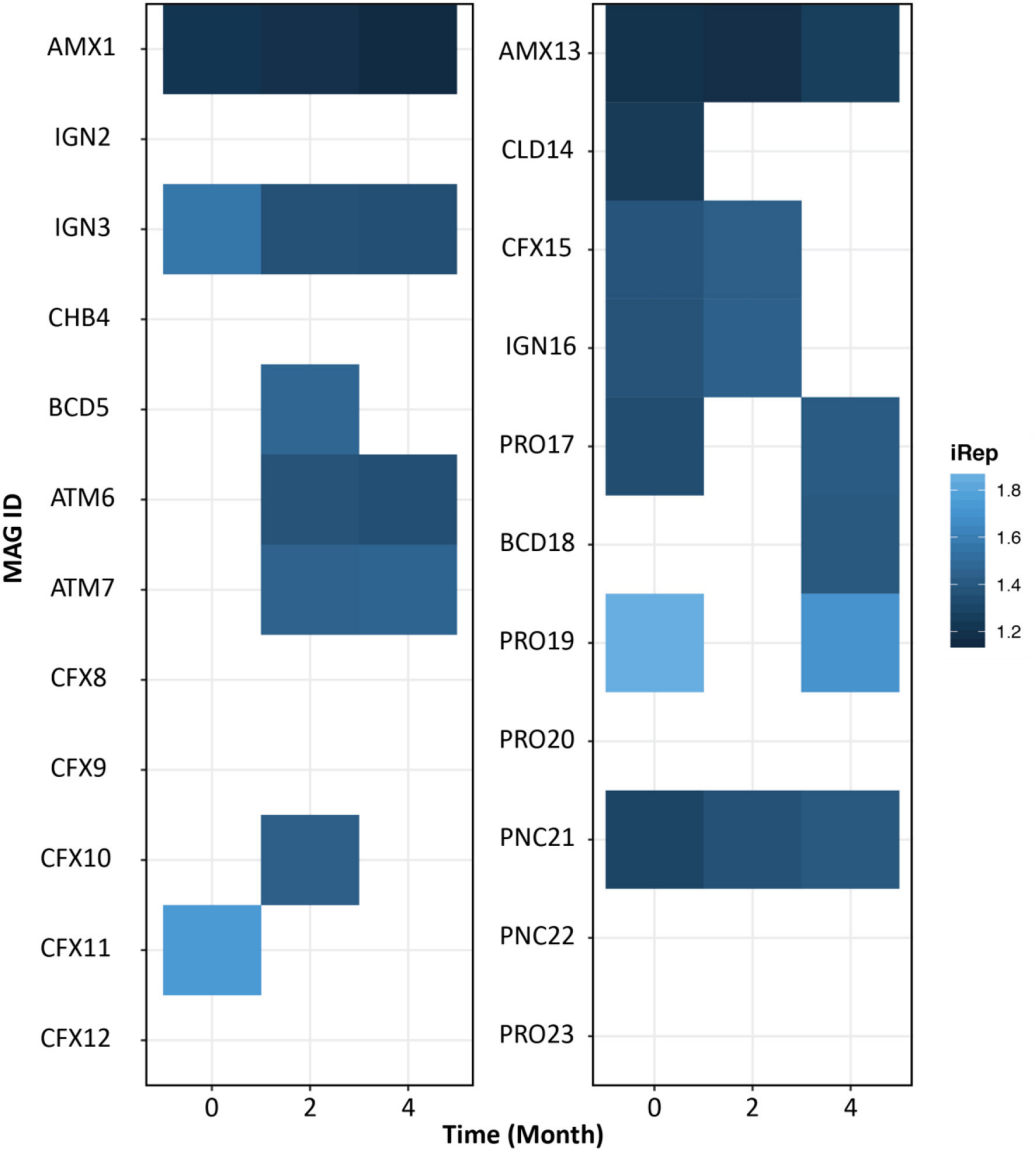
Index of replication (iRep) reflecting bacterial growth rate for freshwater (left) and marine water (right) reactor. Gradient indicates the iRep values. iRep values were estimated for the MAGs with a minimum average coverage of 10 using default parameters. The empty (no-colored) squares represent genomes that did not meet the iRep measurement criteria.

The iRep algorithm was used to understand the growth rate of individual microbiome members. An iRep value of 1, 1.5, or 2 suggests that 0, 50, or 100% of the cells are replicating, respectively (Brown et al., 2016). As expected, we found anammox bacteria growing throughout the operation of both reactors (Figure 5). The bacteria related to phylum *Ignavibacterium* (IGN3) was also growing in the FWR and was also previously reported as the second most dominant phylum in previous studies conducted on non-saline anammox bioreactors (Ali et al., 2016b; Bhattacharjee et al., 2017; Lawson et al., 2017). However, we found PNC21 bacteria constantly growing in MWR, which was also found to be the second most dominant bacterial species in the system.

### A Potential Metabolic Role of Each Population MAG

**Figure 6:**
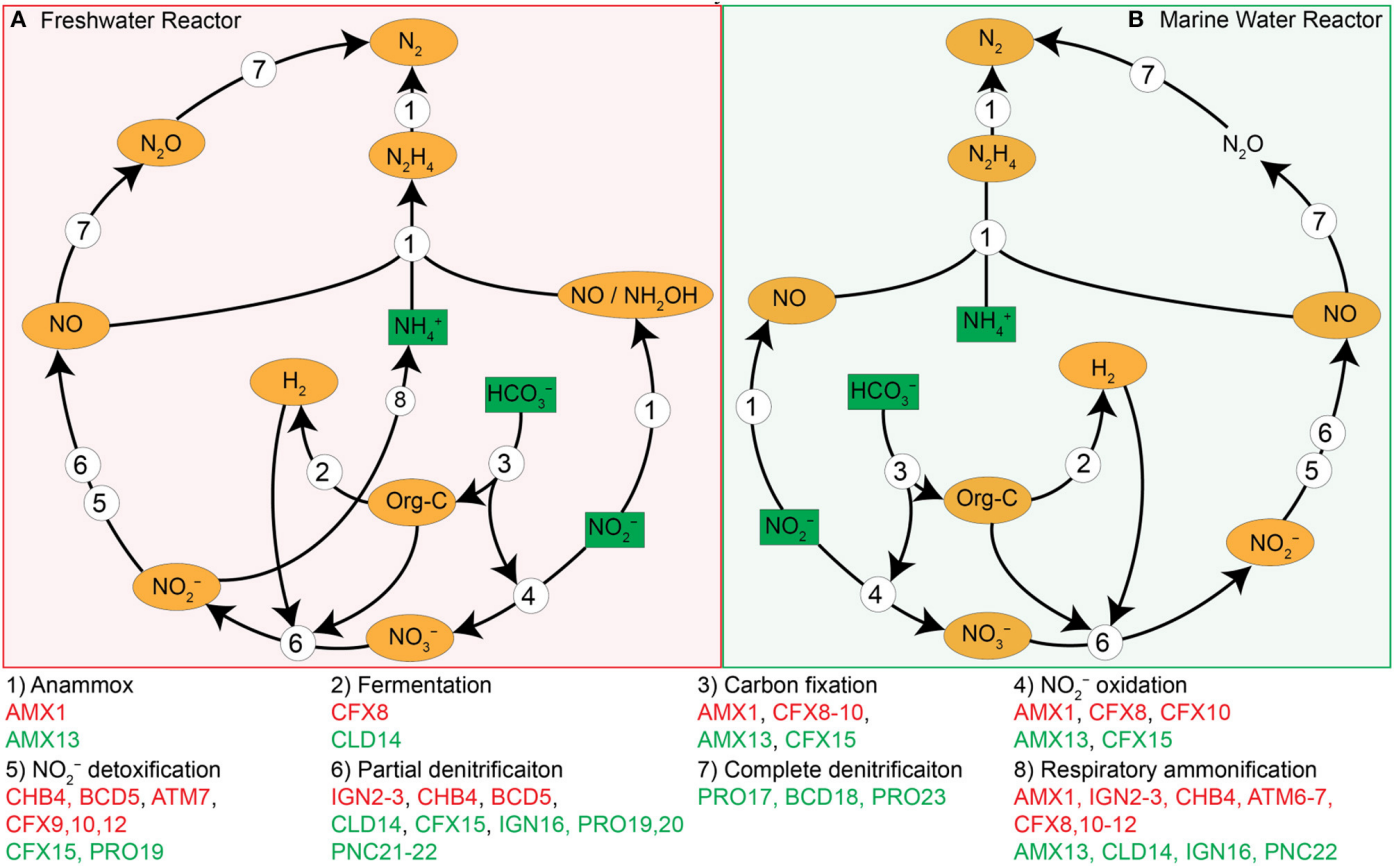
Schematic overview of ecological model in freshwater (**A**, red backdrop) and marine water anammox reactor (**B**, green backdrop). Substrates (${\text{NH}}_{4}^{+}$, ${\text{NO}}_{2}^{-}$, and ${\text{HCO}}_{3}^{-}$) supplied in with the influent are indicated by a green box. Products and intermediates of the bacterial reactions are presented in orange oval. ${\text{NH}}_{4}^{+}$ and ${\text{NO}}_{2}^{-}$ were converted to N_2_ gas by conventional anammox process. Part of ${\text{NO}}_{2}^{-}$ is further oxidized to ${\text{NO}}_{3}^{-}$ by anammox and some other mixotrophic bacteria (CFX8, CFX10, and CFX15) to gain reducing power for CO_2_ fixation through Wood-Ljungdahl pathway. The produced ${\text{NO}}_{3}^{-}$ can be reduced (to ${\text{NO}}_{2}^{-}$, NO, N_2_O, or N_2_) via denitrification with either organic carbon (Org-C) or molecular hydrogen (H_2_) as an electron donor.

A specific ecological role was assigned to each population genome based on the operational conditions, genome content and iRep information, which allowed us to infer a system-wide ecological model for both reactors (Figure 6). The primary substrates supplied in the influent were ${\text{NH}}_{4}^{+}$, ${\text{NO}}_{2}^{-}$, and ${\text{HCO}}_{3}^{-}$ while surplus ${\text{NH}}_{4}^{+}$ and produced ${\text{NO}}_{3}^{-}$ were the primary residues in the effluent, which is typical for anammox-based reactors. As mentioned above, the composition of influent and effluent indicated that the anammox process was mainly contributing toward N-removal in both systems. Based on their genomes, the anammox bacteria (AMX1 and AMX13) and mixotrophs (CFX8-10 and CFX15) would be able to take up CO_2_, which originated from the supplied inorganic carbon (${\text{HCO}}_{3}^{-}$) for assimilation. Autotrophs (AMX1 and AMX13) and mixotrophs (CFX8-10 and CFX15) were the primary suppliers of organic matter in the form of detritus and/or extracellular metabolites such as EPS. The produced organic matter could be utilized by the flanking community of heterotrophs or mixotrophs coupled with some ${\text{NO}}_{3}^{-}$ reduction to ${\text{NO}}_{2}^{-}$, producing additional ${\text{NO}}_{2}^{-}$ available for anammox reaction. The formed CO_2_ either escapes to the atmosphere or is used by bacterial population (AMX1, AMX13, CFX8-10, and CFX15) for biosynthesis. The organic matter could also have been utilized by the fermentative organisms (CFX8, CLD14, and PNC21) as both genomes encoded the enzyme for pyruvate formate-lyase (PFLA) that mediate the conversion of pyruvate to acetyl-CoA and formate. The organism (CFX8) contains the gene (*fdhF*) that encodes for formate dehydrogenase H that facilitates the decomposition of formic acid to H_2_ and CO_2_ under anaerobic conditions in the absence of exogenous electron acceptors. While, the bacterium (PNC21) contains the gene (*fdnH*) encoding for nitrate-inducible formate dehydrogenase that uses formate as an electron donor during anaerobic respiration, coupled with an extracellular ${\text{NO}}_{3}^{-}$ reduction to ${\text{NO}}_{2}^{-}$ (Jormakka et al., 2002). Also, the produced hydrogen could be utilized for autotrophic ${\text{NO}}_{3}^{-}$ reduction to ${\text{NO}}_{2}^{-}$ by the flanking bacterial population (IGN2-3, CFX9, CLD14, and IGN16).

## Discussion

In this study, we operated two (non-saline and saline) lab-scale anammox reactors in parallel treating ${\text{NH}}_{4}^{+}$-rich synthetic wastewater and investigated the genome-resolved microbial ecology of these bioreactors. The microbial community observed in the non-saline reactor (FWR) was similar to the microbial community witnessed in other non-saline anammox reactors reported in previous studies despite the differences in operating conditions, wastewater influent composition, and geographical locations (Speth et al., 2016; Lawson et al., 2017). The dominant anammox bacterium *Ca*. Brocadia sinica was recurrently found as dominant anammox species in many lab-scale (Bhattacharjee et al., 2017; Zhang et al., 2017a) and full-scale anammox reactors (Speth et al., 2016). Previous studies on the kinetics of this anammox bacterium revealed a high maximum specific growth rate (μ_*max*_) (Zhang et al., 2017b) and better aggregation ability (Ali et al., 2018) that could enable this bacterium to outcompete other anammox species (e.g., *Ca*. Jettenia caeni and *Ca*. Kuenenia stuttgartiensis) in engineered systems.

The microbial community in MWR was evidently different from the FWR. Also, the MAG belonging to the phylum Calditrichaeota (CLD14) recovered from MWR was not previously reported in any anammox-based bioreactors. The MAGs belonging to phylum Proteobacteria were only observed in MWR. While the organisms belonging to the phylum Armatimonadetes (ATM6 and ATM7) were only detected in FWR and were actively replicating in the reactor. Bacteria belonging to phylum Armatimonadetes were frequently reported in non-saline ecosystems. A closely related bacterial strain was isolated from a natural freshwater ecosystem that showed optimum growth at high temperature (30–35°C) (Tamaki et al., 2011). Most of anammox reactors are operated at these elevated temperatures, whereas MWR was operated under ambient temperature (~25°C), which could be one of the reasons, other than salinity, for the absence of phylum Armatimonadetes in MWR. It is pertinent to mention, the populations belonging to phylum Planctomycetes (AMX1 in FWR and AMX13 in MWR) were growing during the operation of the reactors (Figure 5).

Anammox bacteria display a large genetic diversity related to nitrite reduction. *Ca*. Scalindua japonica and *Ca*. Kuenenia stuttgartiensis catalyze ${\text{NO}}_{2}^{-}$ to NO with NO-forming cytochrome *cd*_1_-type nitrite reductase (*nirS*) (Kartal et al., 2011; Oshiki et al., 2017). Similarly, a nitrite reductase gene is present in *Ca*. Scalindua brodae genome (Schmid et al., 2003), whereas, the marine anammox bacterium (AMX13) encoded the *nirK*-type copper-containing nitrite reductase, instead of a *nirS*-type for reduction of ${\text{NO}}_{2}^{-}$ to NO. Besides, *Ca*. Jettenia caeni and *Ca*. Brocadia fulgida genome encodes the same nitrite reductase (Gori et al., 2011; Hira et al., 2012). On the contrary, *Ca*. Brocadia sinica and AMX1 genomes lacked the genes *nirS* or *nirK*, pointed toward different ${\text{NO}}_{2}^{-}$ reduction pathway could be adopted by these bacterial strains. Similarly, another anammox bacterium *Ca*. Brocadia sapporoensis genomes, belonging to the same genus *Ca*. Brocadia, also lack nitrite reductase genes (Ali et al., 2016a; Narita et al., 2017). This observation is consistent with the recently proposed hydroxylamine-dependent anammox mechanism in *Ca*. Brocadia sinica that first reduces ${\text{NO}}_{2}^{-}$ to NH_2_OH (instead of NO) by an unknown nitrite reductase, and subsequently converts to NH_2_OH and ${\text{NH}}_{4}^{+}$ to N_2_H_4_ (Oshiki et al., 2016a). The *hao*-like gene, lacking a crosslinking tyrosine in the c-terminus, was expressed by *Ca*. Brocadia sinica bacterium has been proposed to be involved in ${\text{NO}}_{2}^{-}$ reduction reaction (Oshiki et al., 2016a; Lawson et al., 2017). It should be highlighted that a recent work showed compelling evidence for the production of NO (obligate intermediate) by HAO enzyme under both anaerobic and aerobic conditions (Caranto and Lancaster, 2017). It was stated that HAO enzyme catalyzes the oxidation of NH_2_OH by only three electrons to NO, then an unknown enzyme involves further oxidation reaction for the conversion of NO to ${\text{NO}}_{2}^{-}$. Also, recently it was demonstrated that anammox bacteria oxidized ${\text{NH}}_{4}^{+}$ to dinitrogen gas with NH_2_OH as intermediate of the process while transferring electrons to insoluble extracellular electron acceptors such as graphene oxide or electrodes in microbial electrolysis cells (Shaw et al., 2020). Shaw et al. (2020) also suggested a potential HAO enzyme in *Ca*. Brocadia sinica, an ortholog of the proposed nitrite reductase in *Ca*. Kuenenia stuttgartiensis, to be responsible for the four-electron reduction of ${\text{NO}}_{2}^{-}$ to NH_2_OH. In summary, HZS enzyme of both *Ca*. Brocadia sinica and *Ca*. Kuenenia stuttgartiensis could utilize NH_2_OH as a substrate for N_2_H_4_ synthesis, while NH_2_OH is produced differently. *Ca*. Brocadia sinica cells directly reduce ${\text{NO}}_{2}^{-}$ to NH_2_OH, while *Ca*. Kuenenia stuttgartiensis first reduce ${\text{NO}}_{2}^{-}$ to NO (Kartal et al., 2011), and then HZS enzyme mediates reduction of NO by three electrons to NH_2_OH (Dietl et al., 2015). However, the potential HAO enzyme of *Ca*. Brocadia sinica proposed elsewhere (Shaw et al., 2020), should be further investigated as a possible candidate for nitrite reductase.

Although N_2_O is not an intermediate of the anammox process (Kartal et al., 2011), it can be produced abiotically (Kampschreur et al., 2009; Liu et al., 2017) and by other members of the community (Okabe et al., 2011; Ali et al., 2016b). It is more likely that the majority of N_2_O emitted from these systems are derived through the abiotic conversion of NH_2_OH, which is an established intermediate of *Ca*. Brocadia sinica. As pointed in our study, there was no microbial player, except CFX11, in FWR that could mediate N_2_O emission from the system. Although N_2_O is not considered as an intermediate of the anammox process, AMX13 genome contained gene (*norB*) that encodes cytochrome c-dependent nitric oxide reductase (NOR), suggesting that marine anammox has genetic potential to produce N_2_O. Likewise, other population genome PRO20 also encoded the same NOR enzyme, but lacked the downstream reduction *nos* gene, highlighting the genetic potential of MWR microbial community to produce N_2_O emission of N_2_O should be confirmed experimentally in future studies on anammox reactor treating saline wastewater.

Bacteria belonging to phylum *Chloroflexi* encoded the key enzymes required for CO_2_ fixation via the reductive acetyl-CoA pathway. Some members belonging to phylum Chloroflexi were actively replicating in both reactors (Figure 5). Chloroflexi have frequently been detected in anammox-based reactors (Gonzalez-Gil et al., 2014; Ali et al., 2016b). Some members were also reported to possess the same genes of anammox bacteria required for CO_2_ fixation via the reductive acetyl-CoA pathway (Speth et al., 2016). Mixotrophic growth mode would allow these bacteria to thrive under organic limiting conditions, which are typical conditions in anammox reactors treating inorganic ${\text{NH}}_{4}^{+}$-rich wastewater streams.

Our genomic analysis suggests that anammox bacteria growth are more alike prototroph, in which they synthetize their metabolites (i.e., amino acids and vitamins) from inorganic compounds. While, most of the neighboring microbial community are auxotroph and unable to synthesize essential compounds required for growth (e.g., B-vitamin, peptides, and amino acids) and must obtain these compounds through substrate intake. On the other hand, auxotrophs of the Phyla *Ignavibacteriae, Chlorobi*, and *Chloroflexi* were frequently observed as flanking dominant population in anammox reactors (Speth et al., 2016; Lawson et al., 2017). Some of these bacteria were found to encode the respiratory nitrate reductase (*nar*) gene involved in the respiration of ${\text{NO}}_{3}^{-}$ to ${\text{NO}}_{2}^{-}$ (Figure 2B). Therefore, from the genomic information, it is hypothesized that the high abundance and constant presence of these bacteria in the flanking community may suggest a synergy in which the organic matter, nutrients, and metabolites required by the neighboring community members (such as auxotrophs and heterotrophs) are provided by anammox bacteria in the form of dead biomass or EPS of the biofilm. While the flanking community members couple oxidation of organic carbon with the reduction of nitrate to nitrite, which is a substrate for the anammox reaction. In fact, bacteria affiliated to phylum *Ignavibacteriae* were reported as the second most dominant in non-saline anammox reactors and play a role to catabolize extracellular peptides bound in the EPS matrix, while respiring nitrate (to nitrite) (Lawson et al., 2017). Notably, bacteria belong to genome IGN3 were found actively growing. On the other hand, bacteria affiliated with phylum Planctomycetes (family *Planctomycetaceae*) were consistently found to be the second most dominant population in MWR. This bacterium may play a similar role, degrading detritus and extracellular peptide while respiring extracellular ${\text{NO}}_{3}^{-}$ to ${\text{NO}}_{2}^{-}$ to provide the substrate to anammox bacteria. In anammox-based biofilm structure (either granular or attached biofilm), ${\text{NO}}_{2}^{-}$ (electron acceptor) could be the limiting substrate in the inner layer as compared to ${\text{NH}}_{4}^{+}$ (electron donor), as ${\text{NH}}_{4}^{+}$ has higher effective diffusion coefficient, as compared to ${\text{NO}}_{2}^{-}$, allowing it to diffuse/penetrate deeper and faster in the biofilm (Stewart, 2003). Therefore, anammox bacteria might develop a commensalism/mutualism with neighboring microbes to obtain the electron acceptor (i.e., ${\text{NO}}_{2}^{-}$ and NO), and in return, these adjacent microbes catabolize extracellular material bound in the anammox EPS matrix.

This study provided a detailed insight into the microbial community structure and their role in two different (non-saline and saline) engineered anammox ecosystems. Despite the different microbial communities in the reactors, there was functional redundancy in both ecosystems. Therefore, since the genetic potential is analogous, marine anammox bioreactors could be utilized for nitrogen removal from moderate to high-saline wastewaters for industrial and domestic applications—where seawater is used for toilet flushing (Ali et al., 2020). Thus, functional redundancy may play an important role in the functional resistance of anammox bacteria to treat saline wastewater generated from toilet flushing having variable salinity. This would represent some advantages over non-saline anammox since marine anammox species present a higher affinity for ammonium and work at lower temperatures, conditions that are typical in domestic wastewater (Awata et al., 2013). Seawater is used to dilute toxic pollutants in industrial sewage to make it biologically treatable. The tolerance to high salinity by marine anammox bacteria, make it suitable for wastewater treatment of some industrial effluents such as fish canning, seafood processing, tanning industry, etc. The metabolic pathways depicted in this study, illustrate the microbial ecology of the community in enriched anammox reactors. The high abundance of heterotrophic organisms in FWR and MWR investigated here suggests that they play an ecological role in community function, and their contribution should be examined in future studies. Finally, a system-wide ecological model was constructed based on the putative role of each community member in both bioreactors. It should be further validated using, for instance, metatranscriptomics, metaproteomics, or enrichment/isolation of critical organisms from this reactor.

## Data Availability Statement

All sequencing data are under BioProject PRJNA592266 in NCBI.

## Author Contributions

MAli, DS, and PS conceived the study. MAli, DS, and MAlb analyzed the data. All authors contributed significantly in the writing of the manuscript.

## Conflict of Interest

MAlb is a co-founder of the sequencing company DNASense ApS. The remaining authors declare that the research was conducted in the absence of any commercial or financial relationships that could be construed as a potential conflict of interest.
