# Supplementary material for: Comparative Genome-Centric Analysis of Freshwater and Marine ANAMMOX Cultures Suggests Functional Redundancy in Nitrogen Removal Processes
Source: Front Microbiol. 2020 Jul 7;11:1637. doi: 10.3389/fmicb.2020.01637 (PMC7358590; doi:10.3389/fmicb.2020.01637)
Supplement: Supplementary file 1 [file Data_Sheet_1.PDF]

Supplementary Material

1 Supplementary Figures and Tables

1.1 Supplementary Tables

Table S1: Operating conditions of both anammox reactors

Table S2: Nitrogen cycle marker genes

Table S1: Operating conditions of anammox reactors used in this study.

Parameters	Freshwater Anammox	Marine Water Anammox
**Dominant species**	*Ca.* Brocadia sinica	*Ca.* Scalindua
**Biofilm type**	Granular biomass	Biofilm attached to non-woven sheet
**Operating temperature**	37°C	Ambient (~22 °C)
**Feed water**	Deionized water	Red Sea water
**Salinity**	< 0.1%	~ 3.5%
**NH_4^+ concentration**	2.5–20 mM	2.5–5.0 mM
**NO_2^- concentration**	2.5–22.5 mM	2.5–5.0 mM
**Hydraulic retention time**	0.15 day	0.46 day
**Nitrogen loading rate**	~ 4.0 kg–N m^{-3} d^{-1}	~ 0.3 kg–N m^{-3} d^{-1}

Table S2: Nitrogen cycle marker genes

Genes	Protein	KO Number	Enzyme
narG, narZ, nxrA	nitrate reductase / nitrite oxidoreductase, alpha subunit	K00370	EC:1.7.5.1–1.7.99.-
narH, narY, nxrB	nitrate reductase / nitrite oxidoreductase, beta subunit	K00371	EC:1.7.5.1–1.7.99.-
narI, narV	nitrate reductase gamma subunit	K00374	EC:1.7.5.1–1.7.99.-
napA	periplasmic nitrate reductase NapA	K02567	EC:1.7.99.-
napB	cytochrome c-type protein NapB	K02568	
nirB	nitrite reductase (NADH) large subunit	K00362	EC:1.7.1.15
nirD	nitrite reductase (NADH) small subunit	K00363	EC:1.7.1.15
nrfA	nitrite reductase (cytochrome c-552)	K03385	EC:1.7.2.2
nrfH	cytochrome c nitrite reductase small subunit	K15876	
narB	ferredoxin-nitrate reductase	K00367	EC:1.7.7.2
nasA	assimilatory nitrate reductase catalytic subunit	K00372	EC:1.7.99.-
norB	nitric oxide reductase subunit B	K04561	EC:1.7.2.5
norC	nitric oxide reductase subunit C	K02305	
nosZ	nitrous-oxide reductase	K00376	EC:1.7.2.4
hao	hydroxylamine dehydrogenase	K10535	EC:1.7.2.6
hdh	hydrazine dehydrogenase	K20935	EC:1.7.2.8
hzs	hydrazine synthase subunit	K20932	EC:1.7.2.7
nirK	nitrite reductase (NO-forming)	K00368	EC:1.7.2.1
nirS	nitrite reductase (NO-forming) / hydroxylamine reductase	K15864	EC:1.7.2.1, 1.7.99.1

1.2 Supplementary Figures

Figure S1: Schematic diagram of the freshwater and marine water anammox reactors.

Figure S2: Time courses of nitrogen loading rates (NLRs, filled circles) and removal rates (NRRs, empty circles) of the freshwater (A) and marine water (B) anammox reactors. Concentrations of NH_4^+ (filled circles) and NO_2^- (filled triangle) in influent and concentrations of NH_4^+ (empty circles), NO_2^- (empty triangles) and NO_3^- (empty diamond) in effluent shown as panel (C) for freshwater and (D) for marine water anammox reactors. Concentrations of NO_3^- in influent was always below 5 mg-N L^{-1}. Operating conditions of these reactors are mentioned in Table S1.

Figure S3: Differential coverage plot of recovered metagenome-assembled genomes (MAGs) from freshwater anammox reactor. A) Differential coverage plot of the metagenomic scaffolds assembled with the 2nd generation short-read (Illumina) sequencing data. B) Differential coverage plot of the scaffolds assembled through long-read sequencing (Oxford Nanopore) technology corrected with the 2nd generation short-read (Illumina) sequencing data. The size of the circles represents the length of the scaffolds. Colors of the circles represent phylum level taxonomic classification, scaffolds with no color could not be assigned a phylum level classification. The x and y-axes show the sequencing coverage in the samples (log-scaled).

Figure S4: Differential coverage plot of recovered metagenome-assembled genomes (MAGs) from marine anammox reactor. A) Differential coverage plot of the metagenomic scaffolds assembled with the 2nd generation short-read (Illumina) sequencing data. B) Differential coverage plot of the scaffolds assembled through long-read sequencing (Oxford Nanopore) technology corrected with the 2nd generation short-read (Illumina) sequencing data. The size of the circles represents the length of the scaffolds. Colors of the circles represent phylum level taxonomic classification, scaffolds with no color could not be assigned a phylum level classification. The x and y-axes show the sequencing coverage in the samples (log-scaled).

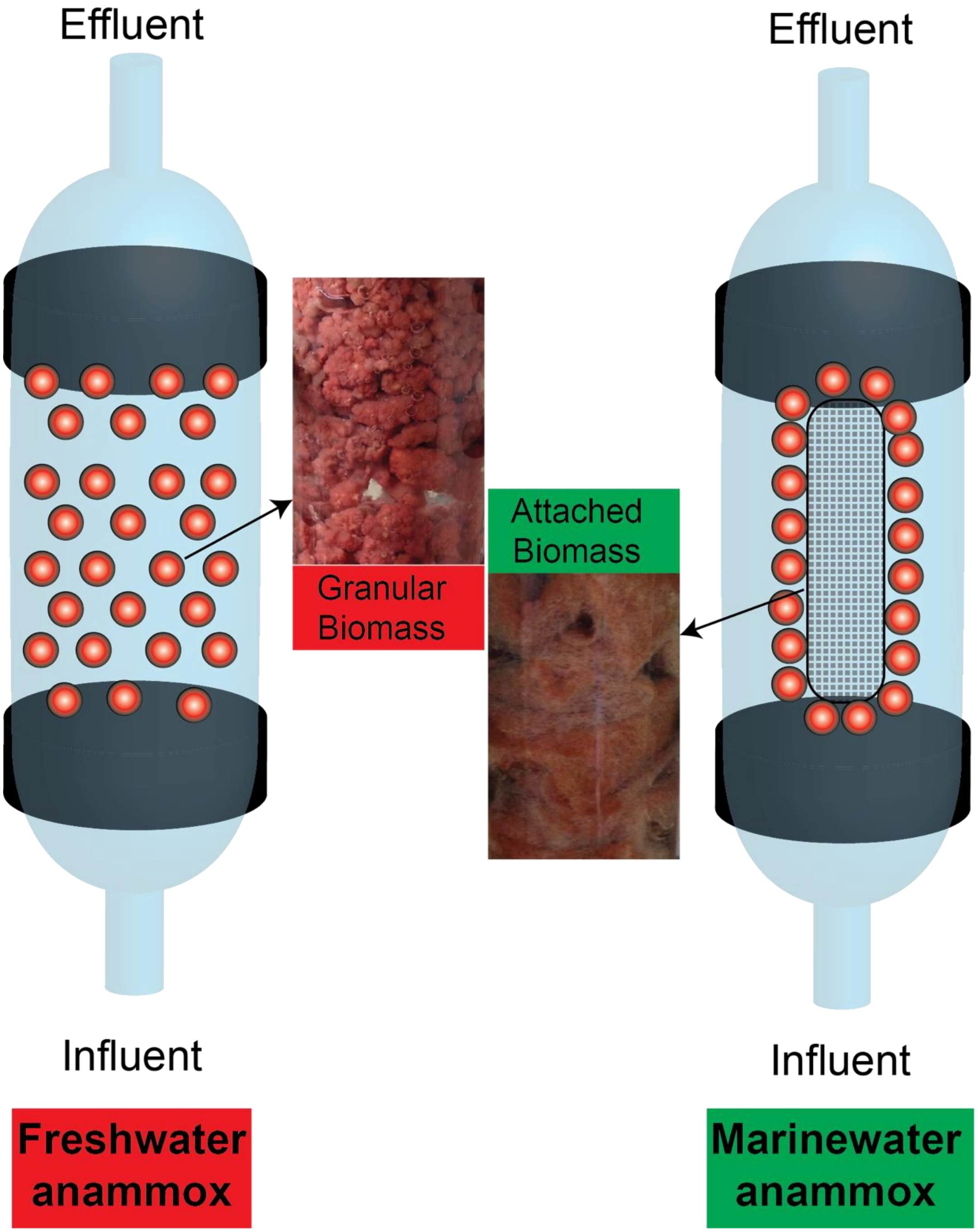

Figure S1: Schematic diagram of the freshwater and marine water anammox reactors used in this study.

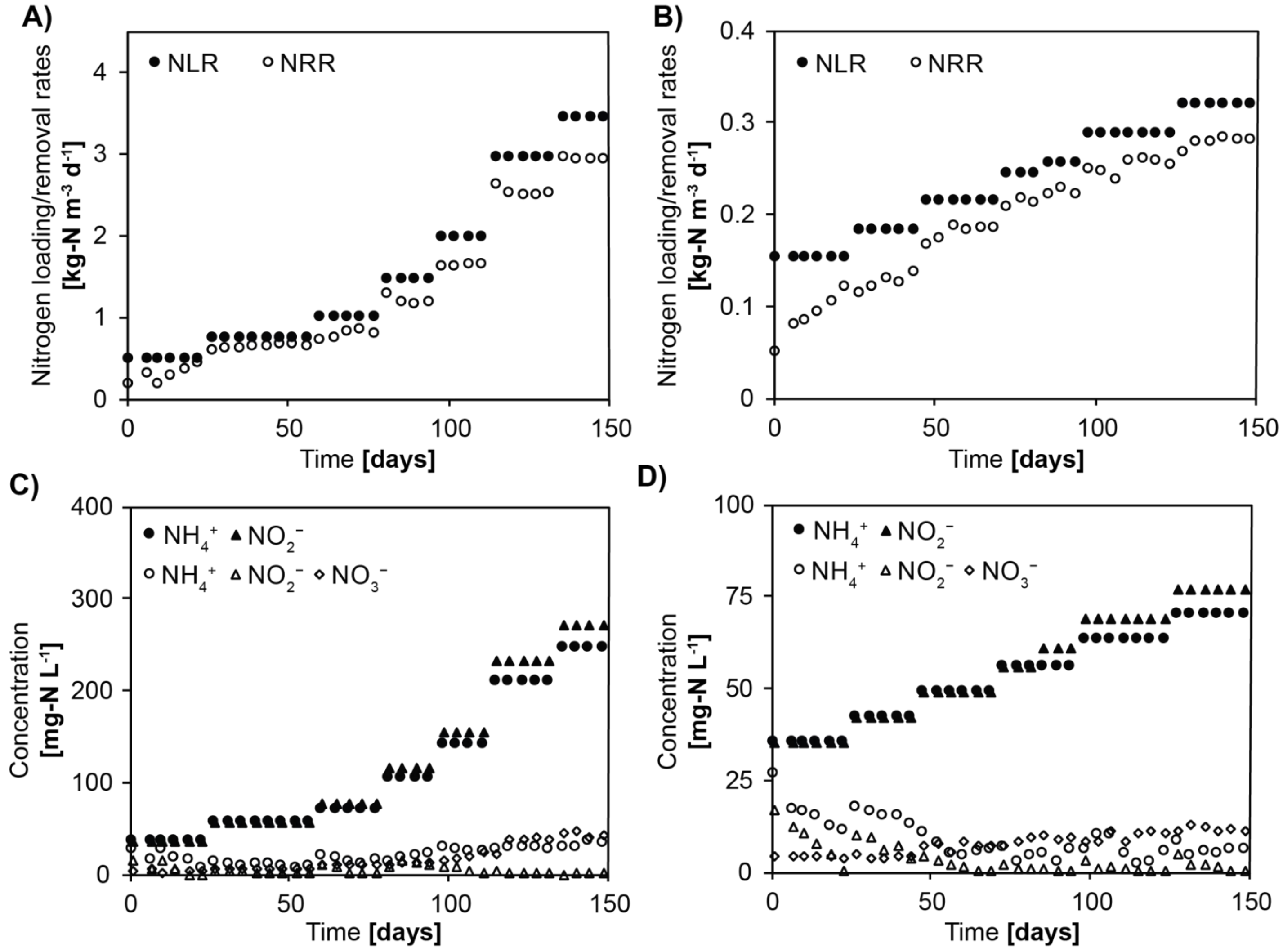

Figure S2: Time courses of nitrogen loading rates (NLRs, filled circles) and removal rates (NRRs, empty circles) of the freshwater **(A)** and marine water **(B)** anammox reactors. Concentrations of NH_4^+ (filled circles) and NO_2^- (filled triangle) in influent and concentrations of NH_4^+ (empty circles), NO_2^- (empty triangles) and NO_3^- (empty diamond) in effluent shown as panel **(C)** for freshwater and **(D)** for marine water

anammox reactors. Concentrations of NO_3^- in influent was always below 5 mg-N L^{-1}. Operating conditions of these reactors are mentioned in **Table S1**.

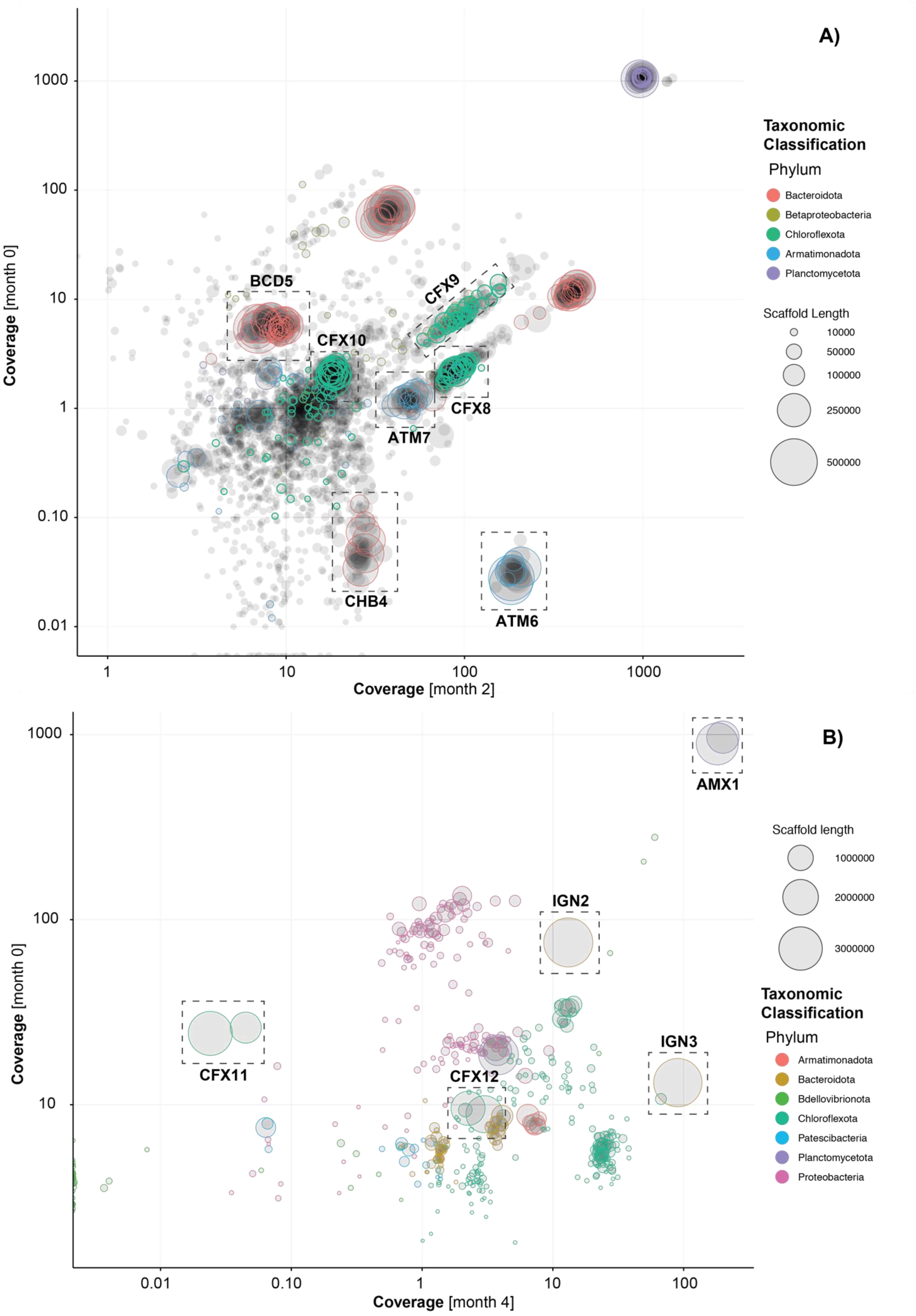
A)
Coverage [month 0]
Coverage [month 2]
1000
100
10
1
0.10
0.01
1
10
100
1000
BCD5
CFX9
CFX10
CFX8
ATM7
CHB4
ATM6
Taxonomic Classification
Phylum
Bacteroidota
Betaproteobacteria
Chloroflexota
Armatimonadota
Planctomycetota
Scaffold Length
10000
50000
100000
250000
500000
B)
Coverage [month 0]
Coverage [month 4]
1000
100
10
0.01
0.10
1
10
100
AMX1
IGN2
CFX11
CFX12
IGN3
Scaffold length
1000000
2000000
3000000
Taxonomic Classification
Phylum
Armatimonadota
Bacteroidota
Bdellovibrionota
Chloroflexota
Patescibacteria
Planctomycetota
Proteobacteria

Figure S3: Differential coverage plot of recovered metagenome-assembled genomes (MAGs) from freshwater anammox reactor. A) Differential coverage plot of the metagenomic scaffolds assembled with the 2nd generation short-read (Illumina) sequencing data. B) Differential coverage plot of the scaffolds assembled through long-read sequencing (Oxford Nanopore) technology corrected with the 2nd generation short-read (Illumina) sequencing data. The size of the circles represents the length of the scaffolds. Colors of the circles represent phylum level taxonomic classification, scaffolds with no color could not be assigned a phylum level classification. The x and y-axes show the sequencing coverage in the samples (log-scaled).

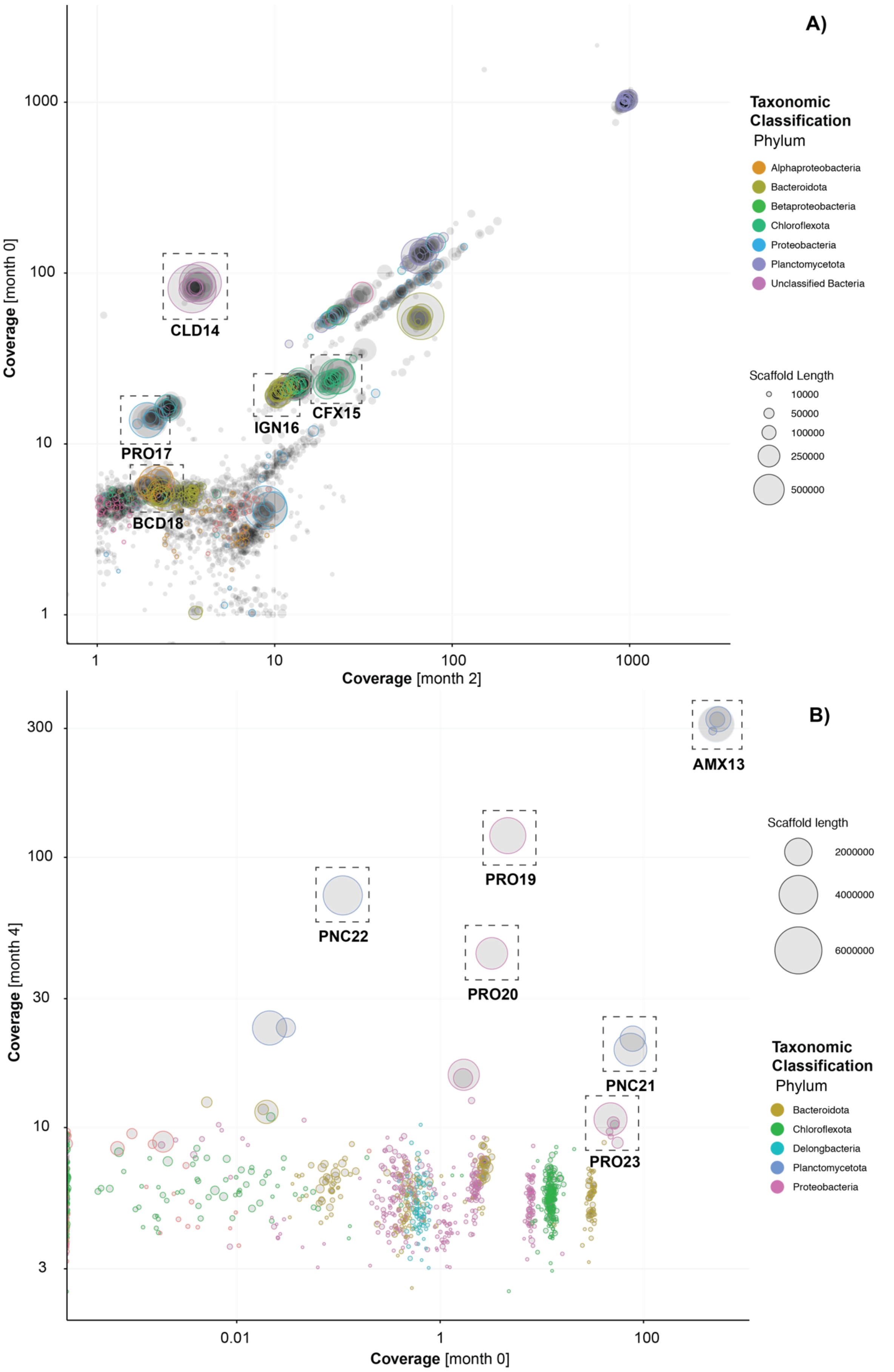
A)
Coverage [month 0]
Coverage [month 2]
1000
100
10
1
1
10
100
1000
CLD14
PRO17
IGN16
CFX15
BCD18
Taxonomic Classification
Phylum
Alphaproteobacteria
Bacteroidota
Betaproteobacteria
Chloroflexota
Proteobacteria
Planctomycetota
Unclassified Bacteria
Scaffold Length
10000
50000
100000
250000
500000
B)
Coverage [month 4]
Coverage [month 0]
300
100
30
10
3
0.01
1
100
AMX13
PRO19
PNC22
PRO20
PNC21
PRO23
Scaffold length
2000000
4000000
6000000
Taxonomic Classification
Phylum
Bacteroidota
Chloroflexota
Delongbacteria
Planctomycetota
Proteobacteria

Figure S4: Differential coverage plot of recovered metagenome-assembled genomes (MAGs) from marine anammox reactor. A) Differential coverage plot of the metagenomic scaffolds assembled with the 2nd generation short-read (Illumina) sequencing data. B) Differential coverage plot of the scaffolds assembled through long-read sequencing (Oxford Nanopore) technology corrected with the 2nd generation short-read (Illumina) sequencing data. The size of the circles represents the length of the scaffolds. Colors of the circles represent phylum level taxonomic classification, scaffolds with no color could not be assigned a phylum level classification. The x and y-axes show the sequencing coverage in the samples (log-scaled).
